# Literary Notes

**Published:** 1903-08

**Authors:** 


### LITERARY NOTES.

THE *Journal of Infectious Diseases* will be issued in the fall, edited by Professor Ludwig Hektoen and Professor Edward O. Jordan of the University of Chicago. The expenses, estimated at $5,000 annually, will be met by Mr. and Mrs. Harold McCormick. Since the death, a few years ago, of their son, Jack Rockefeller McCormick, Mr. and Mrs. McCormick have founded the institute for the study of infectious diseases, connected with Rush Medical College. The *Journal* will give to the scientific world the results of work done there and in other laboratories.

---

THE Arlington Chemical Co. has arranged to issue, under the title "The Law and the Doctor," two 48-page booklets, which shall present in condensed form and succinct style, an epitome of the essentially important features of (1) "The civil liability of the physician for malpractice," and (2) "The physician as a witness." These exceedingly practical monographs have been expressly prepared by an eminent member of the New York Bar, who is well recognised by the legal profession as an expert in this special branch of practice. The first of these reference text manuals is now ready for distribution and after a reasonable interval will be followed by the second monograph. Copies may be had by applying to the above company.

